# Simultaneous Identification of Unroofed Coronary Sinus Atrial Septal Defect and Atrial Septal Defect Secundum Using Cardiac Computed Tomography Angiography

**DOI:** 10.7759/cureus.52575

**Published:** 2024-01-19

**Authors:** Islam A Ibrahim, Mohannad T Howladar, Tahani S Alsaery, Ashraf Anwar, Waqar Ahmad

**Affiliations:** 1 Adult Cardiology, King Fahd Armed Forces Hospital, Jeddah, SAU

**Keywords:** cardiac cta, transesopageal echocardiography, transthoracic echocardiography, unroofed coronary sinus, atrial septal defect

## Abstract

Unroofed coronary sinus (UCS) represents a rare subtype of atrial septal defect (ASD), an adult congenital heart disease characterized by communication between the systemic and pulmonary circulations at the atrial level. This case report presents the unique occurrence of a large, unrepaired ASD secundum and an incidentally identified UCS type II in a 25-year-old female during a cardiac murmur assessment. The diagnosis of ASD secundum was initially made using transthoracic echocardiography (TTE) and was later confirmed with a transesophageal echocardiogram. The identification of the UCS was achieved through the utilization of cardiac computed tomography angiography (CCTA). Ultimately, the patient underwent a successful reroofing procedure using a bovine pericardial patch.

## Introduction

Unroofed coronary sinus (UCS) is a rare subtype of atrial septal defect (ASD), an adult congenital heart disease characterized by communication between the systemic and pulmonary circulations at the atrial level [1]. This condition occurs when there is a partial or complete loss of the coronary sinus wall, resulting in oxygenated blood from the left atrium being carried to the right atrium through the coronary sinus [2]. Left-to-right shunts can lead to heart failure due to volume overload. The clinical impact of these shunts depends on their size, the volume of blood flow, the pressure gradient, and the pulmonary and systemic vascular resistance. Significant shunts occurring above the level of the tricuspid valve, such as ASDs, can lead to right atrial and ventricular enlargement due to increased filling pressure and end-diastolic volume. Conversely, significant shunts below the level of the tricuspid valve, like ventricular septal defects (VSD), can cause left atrial and ventricular enlargement, and may also increase pulmonary artery (PA) flow and pressure [3]. ASD accounts for up to 30% of adult congenital heart disease diagnoses. UCS is the least common type of ASD and is often associated with persistent left superior vena cava (PLSVC) [1]. The incidence of UCS is estimated to be less than 1% of all ASD [1]. Anatomically, UCS-ASD is classified into four groups, according to Kirklin and Barratt-Boyes. Types I and II describe a completely unroofed coronary sinus with or without PLSVC, respectively. Types III and IV describe partially unroofed mid or terminal segments, respectively [4]. The prevalence and incidence of UCS-ASD in Saudi Arabia are currently unknown. Throughout childhood and most of adulthood, UCS remains asymptomatic or presents with mild symptoms. However, complications may arise later in life, such as right heart failure or paradoxical embolism. Diagnosis can be challenging and is often made incidentally during an examination for another condition, most commonly prior to coronary artery bypass surgery [2]. We present a case involving a 25-year-old female patient with the rare coexistence of a large, unrepaired ASD secundum and UCS type II, which were incidentally identified during a cardiac murmur assessment. The ECG showed sinus rhythm, right axis deviation, and a complete right bundle branch block (RBBB). The diagnosis of the ASD secundum was made using transthoracic echocardiography (TTE). Although transesophageal echocardiography (TEE) confirmed the presence of the secundum-type ASD, it did not identify the coexistence of UCS. However, cardiac computed tomography angiography (CCTA) revealed a large UCS-ASD type II located inferiorly. The patient underwent surgical treatment using a bovine pericardial patch.

## Case presentation

**Figure 1:**
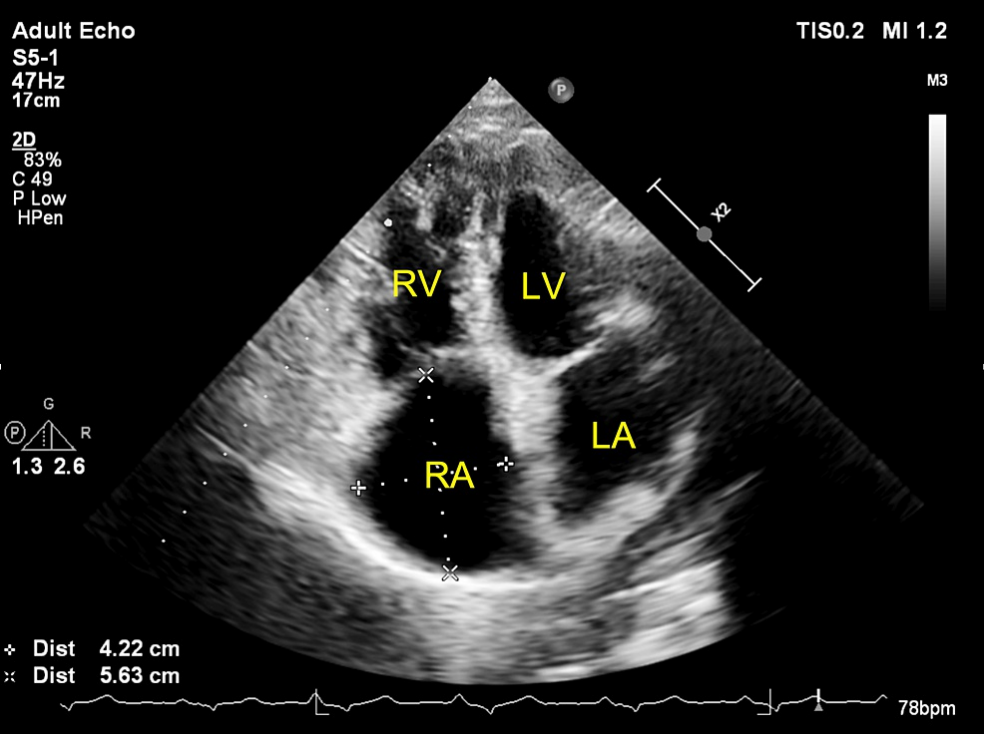
Two-dimensional TTE, apical 4-chamber view, systolic phase, demonstrates a dilated RA. RA: Right Atrium; LA: Left Atrium; RV: Right Ventricle; LV: Left Ventricle.

**Figure 2:**
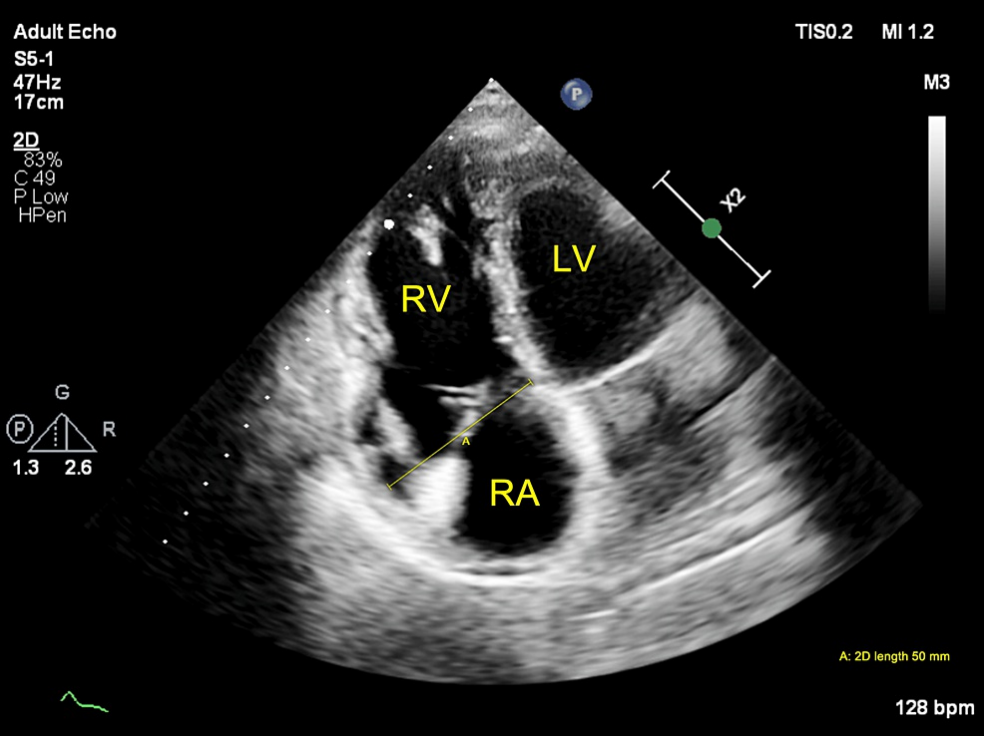
Two-dimensional TTE, apical 4-chamber view, diastolic phase, demonstrates a dilated RV with a basal diameter of 50 mm. TTE: Transthoracic echocardiography; RV: Right ventricle.

**Figure 3:**
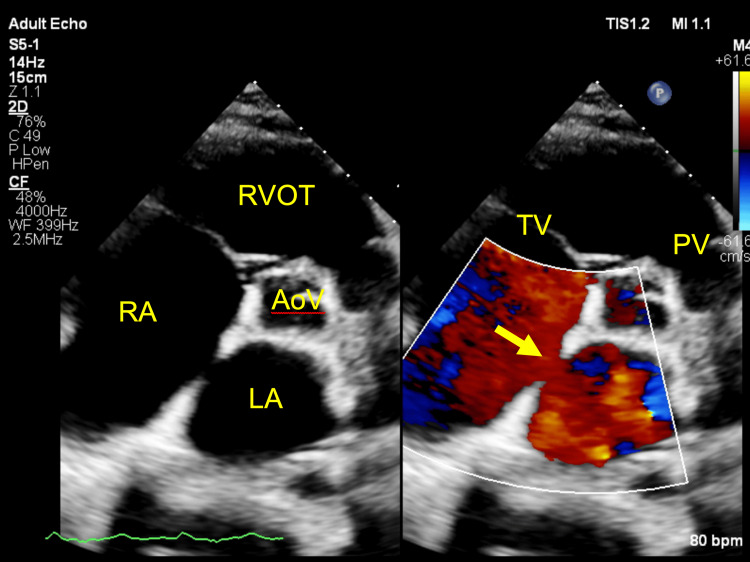
Two-dimensional TTE, parasternal short-axis, aortic valve level, demonstrates a dilated RA and a large ASD of the secundum type (arrow) with left-to-right flow by color Doppler. RVOT: Right Ventricle Outflow Tract; AoV: Aortic Valve; TV: Tricuspid Valve; PV: Pulmonary Valve; TTE: transthoracic echocardiography; ASD: Atrial septal defect; RA: Right atrium.

**Figure 4:**
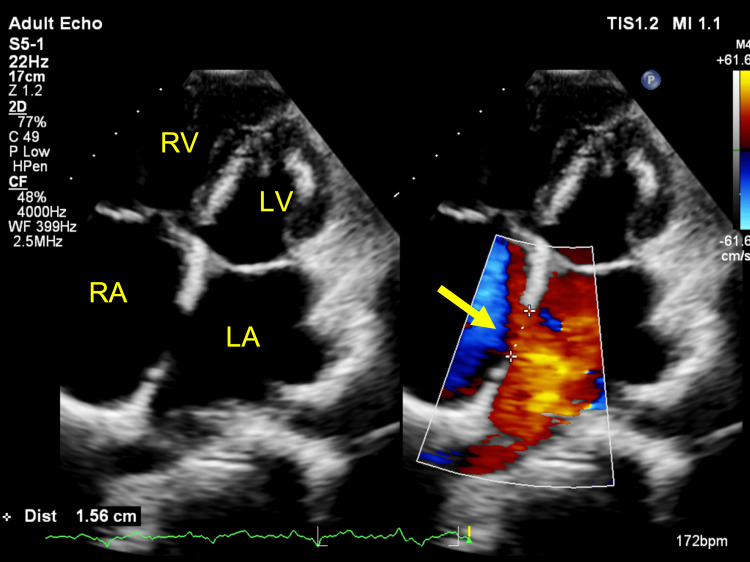
Two-dimensional TTE, apical 4-chamber view with color flow Doppler, systolic phase, demonstrates a large ASD (arrow) with a longitudinal diameter of 15.6 mm. TTE: Transthoracic echocardiography; ASD: Atrial septal defect.

**Figure 5:**
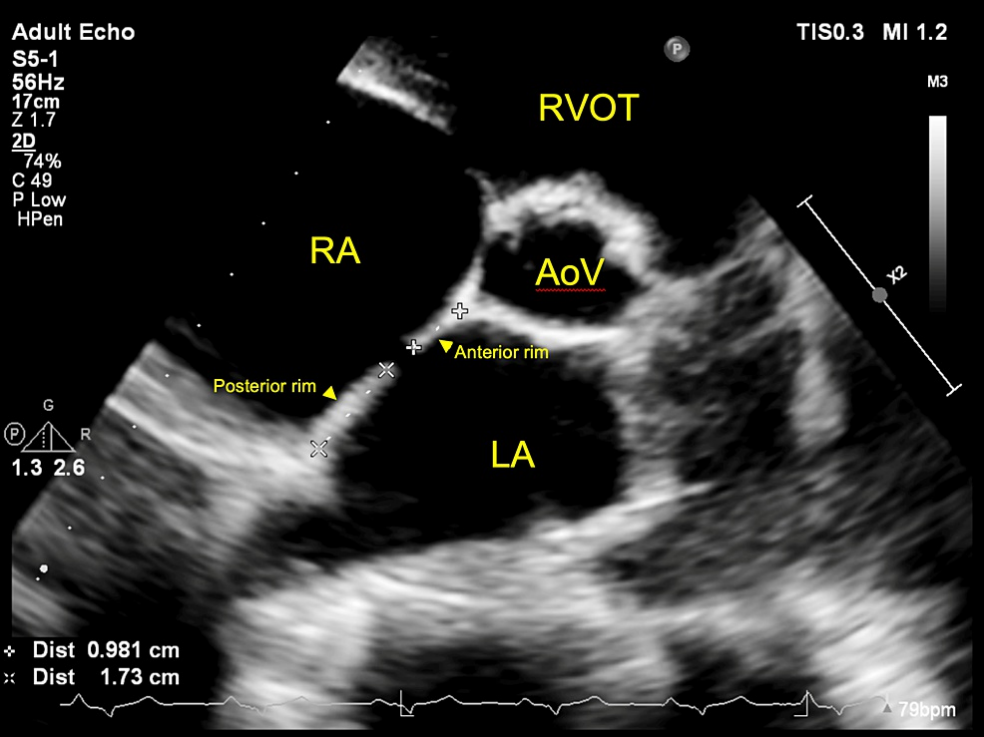
Two-dimensional TTE, parasternal short-axis, aortic valve level, demonstrates the measured anterior and posterior ASD secundum defect. TTE: Transthoracic echocardiography; ASD: Atrial septal defect.

**Figure 6:**
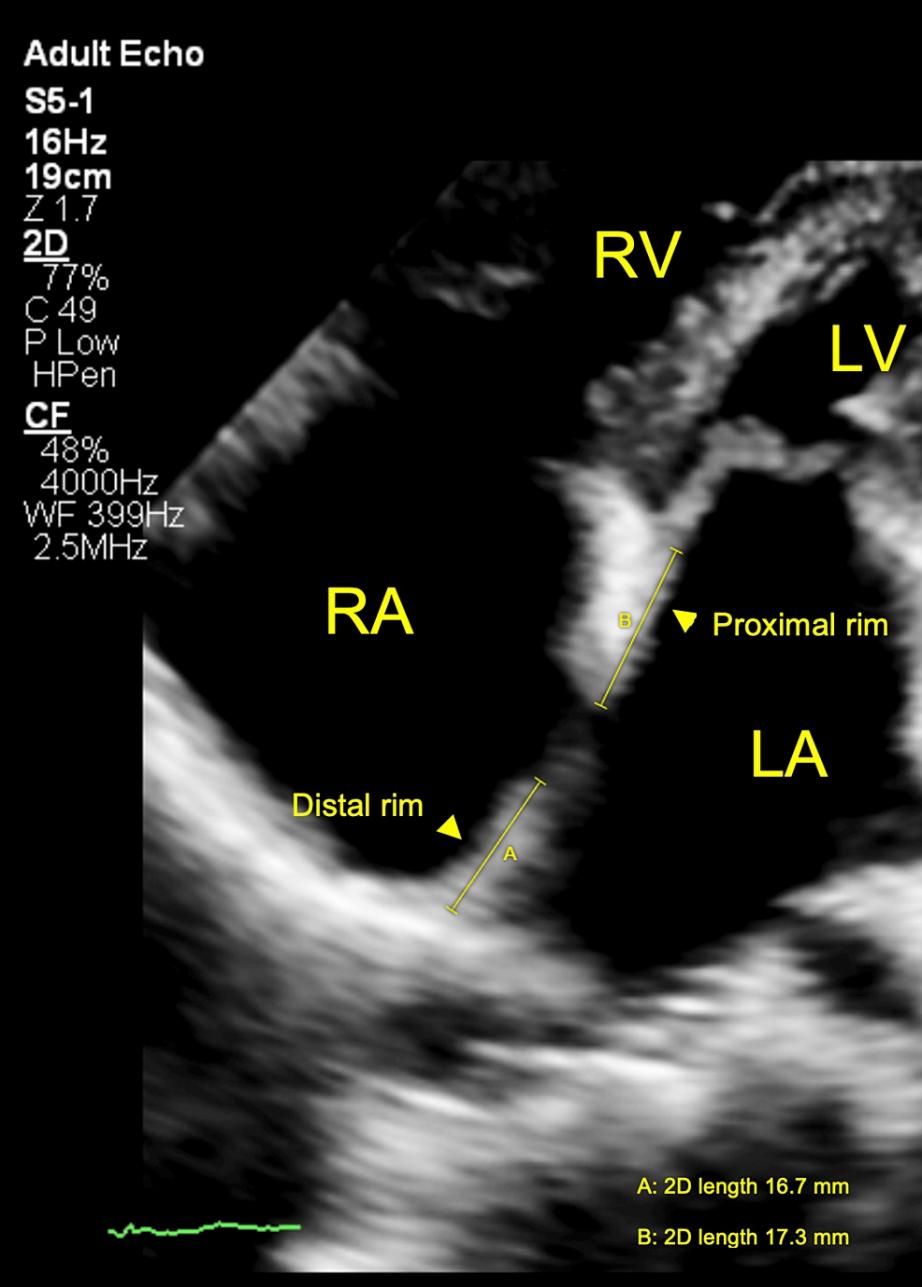
Two-dimensional TTE, apical four-chamber view, systolic phase, demonstrates a large ASD with both proximal (distance B) and distal (distance A) rims measuring approximately 17 mm each. TTE: Transthoracic echocardiography; ASD: Atrial septal defect.

A previously healthy 25-year-old female presented with flu-like symptoms and was found to have an ejection systolic murmur upon auscultation. She had no history of dyspnea or palpitations during her childhood. As part of her evaluation, a chest X-ray revealed cardiomegaly. Subsequent TTE showed dilated atria, a dilated right ventricle, and an interatrial shunt. Consequently, she was referred to our facility for further assessment. On auscultation, a loud ejection systolic murmur III/VI that intensifies with inspiration was best heard at the left second intercostal space. The second heart sound (S2) had a prominent pulmonic valve component (P2) and showed widely fixed splitting. Her oxygen saturation level was 98%. An ECG demonstrated sinus rhythm with right axis deviation and complete RBBB. A repeat TTE at our facility confirmed the dilation of the right atrium (RA) and right ventricle (RV), with the RV basal diameter measuring 50 mm (Figures 1-2). Additionally, an ASD of the secundum type measuring approximately 15 mm was observed at the mid-interatrial septum (IAS), with left-to-right flow indicated by color Doppler imaging and a QP/QS ratio of 3.1 (Figures 3-4). The anterior rim measured 8 to 9 mm thick; the posterior rim, 18 mm thick; the distal rim, 17 mm thick; and the proximal rim, 17 mm thick (Figures 5-6).

**Figure 7:**
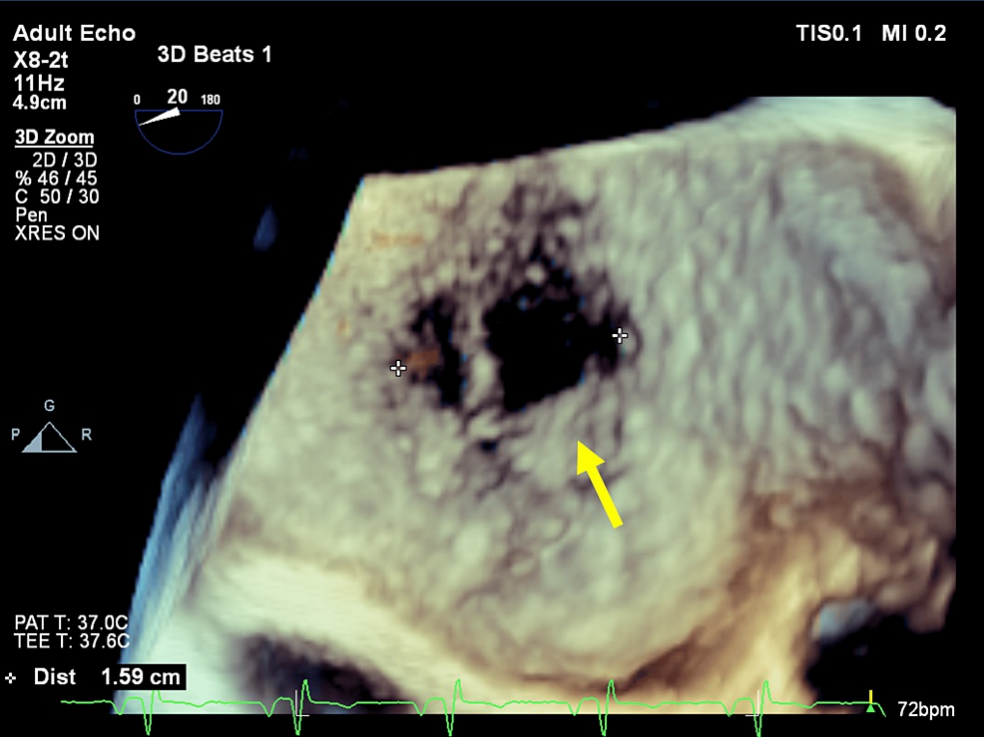
Three-dimensional TEE, mid-esophageal level, and four-chamber view (20o) demonstrate the large fenestrated ASD secundum type. TTE: Transthoracic echocardiography; ASD: Atrial septal defect.

TEE showed a markedly dilated left atrium (LA). There was a fenestrated defect at the mid-IAS with two left-to-right flow jets, the larger of which measured 8 mm x 8 mm and the smaller 5 mm x 8 mm, separated by a thin membrane (Figure 7). The total defect diameter was 15 mm x 8 mm, the rim thickness was 19 mm proximally and distally, the anterior rim was 8 mm thick, the posterior rim was 13 mm thick, and the superior and inferior rims were 12 mm and 14 mm thick, respectively. The ASD resembled Swiss cheese at 1 o'clock with a band in the middle, measuring 8 x 8 mm and 8 x 5 mm, approximately 15 to 16 mm in diameter. She exhibited significant RV and RA dilatation, with a Qp/Qs ratio of 3.0.

**Figure 8:**
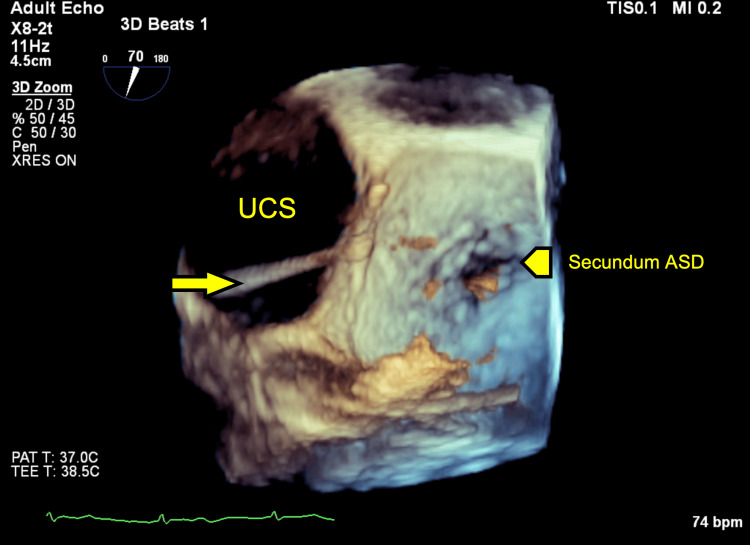
Intraprocedural 3D TEE, LA aspect view, diastolic phase, showing the IAS with ASD secundum (arrowhead) and demonstrating a multipurpose catheter (arrow) passing through the UCS. TEE: Transesophageal echocardiography; LA: Left atrium; IAS: Interatrial septum; ASD: Atrial septal defect

**Figure 9:**
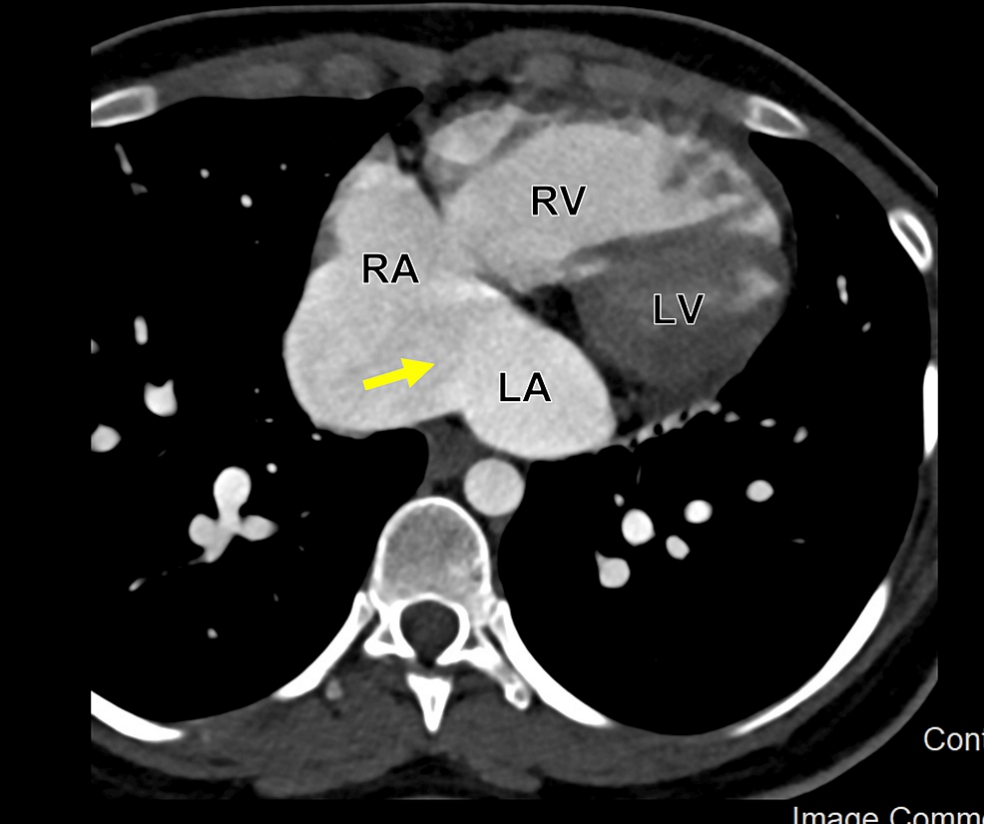
Cardiac computed tomography angiography (CCTA) in axial display showing a communication between the RA and LA representing ASD. RA: Right atrium; LA: Left atrium; ASD: Atrial septal defect.

**Figure 10:**
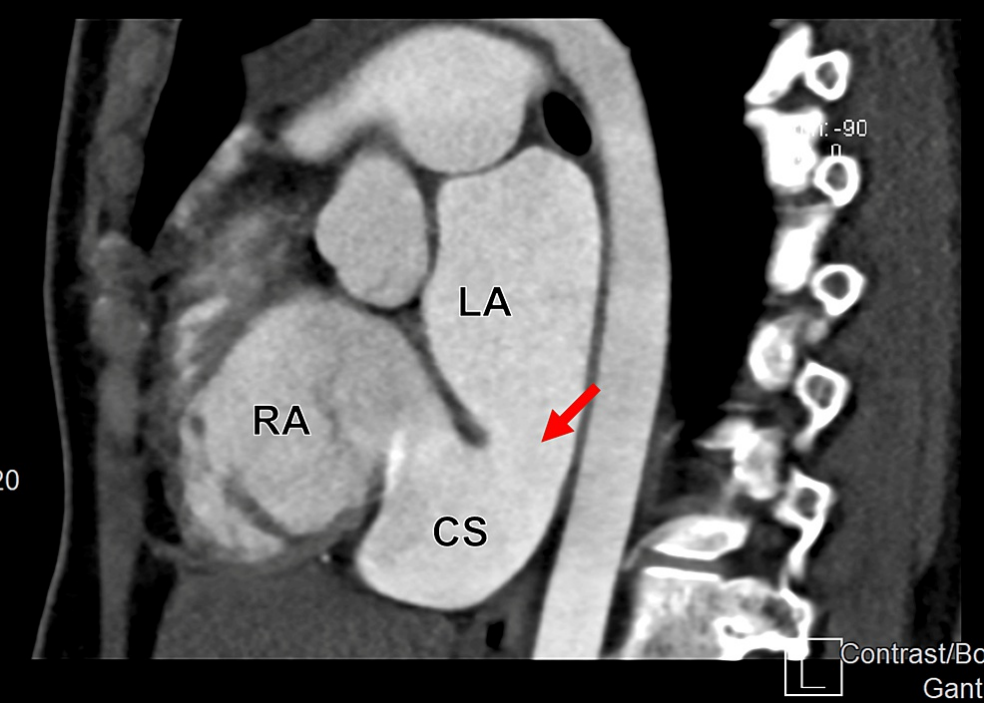
Cardiac computed tomography angiography (CCTA) in a sagittal display showing a communication between the RA and LA, representing an unroofed CS (arrow). RA: Right atrium; LA: Left atrium; CS: Coronary sinus.

**Figure 11:**
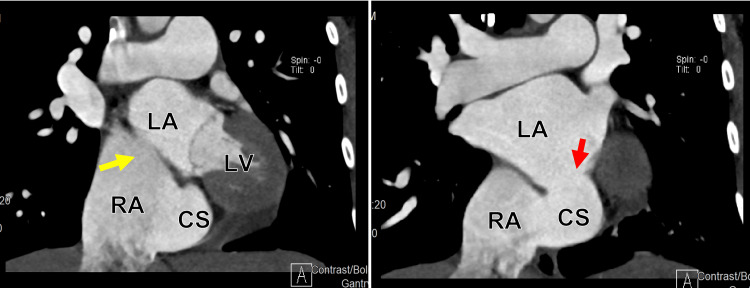
Cardiac computed tomography angiography (CCTA) in sagittal displays showing a dilated RA, an ASD (yellow arrow), and an unroofed CS (red arrow). RA: Right atrium; CS: Coronary sinus; ASD: Atrial septic defect.

**Figure 12:**
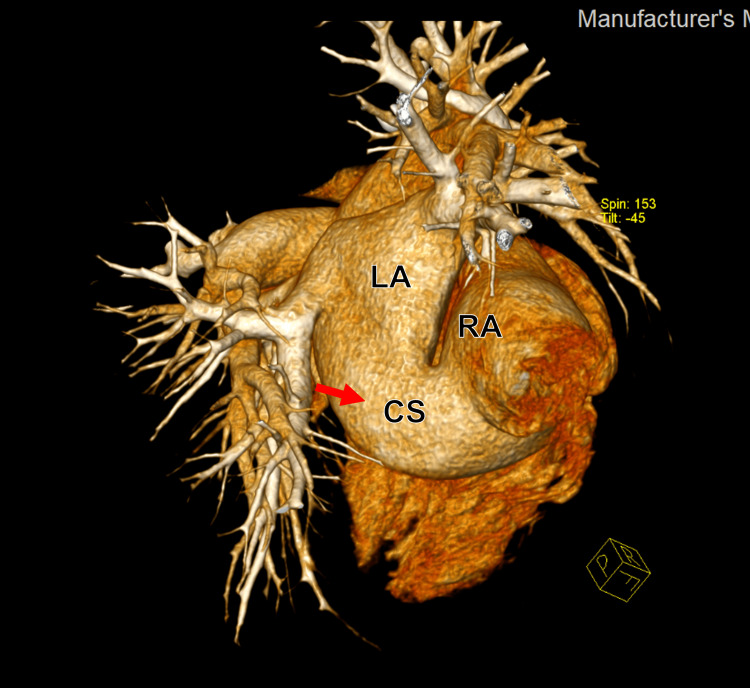
Three-dimensional reconstruction from cardiac computed tomography angiography (CCTA) showing the unroofed and dilated coronary sinus (CS).

The patient was taken to the catheterization laboratory for ASD secundum-type closure using an Amplatzer device under TEE guidance. However, during the procedure, the multipurpose catheter crossed very inferiorly at the level of the coronary sinus (CS) into the LA. The left upper pulmonary vein was easily identified, as was a small CS vein, draining from the great cardiac vein. TEE showed that the multipurpose catheter was in the LA, but it did not go through the previously mentioned secundum ASD, raising the possibility of different co-existing septal defects (Figure 8). The procedure was aborted, and the patient was scheduled for CCTA to better define the septal defect and rule out the presence of PLSVC. CCTA showed a large ASD inferiorly, representing a UCS. The defect was large, measuring 24 mm in length, and both the superior and inferior vena cava were intact. The pulmonary veins drain into the LA. The right pulmonary veins emerged from a single trunk, while the left pulmonary veins emerged from a very short trunk that split into two branches early on (Figures 9-12). The patient was then referred to cardiac surgery services at our facility. She underwent a successful reroofing using a bovine pericardial patch. After the closure, an echocardiogram showed no remaining interatrial shunt or inferior vena cava obstruction, and good biventricular function. She was discharged shortly afterward.

## Discussion

UCS is an infrequent congenital cardiac anomaly that was first described in 1965 by Raghib G et al. [5]. It is characterized by the complete or partial loss of the CS wall, resulting in an IAS between the left and right atria [1]. It is frequently associated with different cardiac abnormalities; the most common is persistent left superior vena cava (PLSVC), which often drains into the LA if present, causing right-to-left shunting. Other known associations include tetralogy of Fallot, tricuspid stenosis, and tricuspid atresia [6, 7]. UCS-ASD diagnosis is quite challenging because most patients are asymptomatic and, if they ever do develop symptoms, they are usually nonspecific. In contrast, as a result of a right-to-left shunt in those with an anomalous PLSVC draining into the LA, cyanosis and polycythemia can occur, resulting in early detection of UCS. TTE can detect an IAS but can easily miss UCS because it is a deep posterior structure. Nevertheless, the presence of an enlarged CS on TTE may suggest the existence of a UCS and/or PLSVC. TEE, on the other hand, can accurately identify UCS in most people. However, CCTA appears to be superior to both TTE and TEE due to its high spatial and temporal resolutions, multiplanar reconstruction capabilities, and large field of view. CCTA can detect and characterize as well as identify related abnormalities of the heart and pulmonary vasculature [1, 8, 9]. The preferred treatment for UCS-ASD is believed to be surgery [1]. The prognosis of surgical repair is favorable, with a mortality rate ranging from 0% to 4%, which is attributed to associated complex congenital heart disease. Shi H et al. reported 159 cases that were surgically repaired. The in-hospital mortality was 3.1% due to the associated severe complex congenital heart disease. A similar outcome was observed in Attenhofer Jost CH et al.'s case series, where they reported 25 repaired cases with single mortality attributed to mediastinitis due to complex congenital heart disease necessitating multiple redo surgeries. In addition, Ootaki Y et al. reported 11 successful reports with no mortality or long-term complications [10, 11]. Percutaneous interventions such as covered stenting or implantation of Amplatzer occluding devices have shown encouraging outcomes, but further research is required before they can be implemented into routine practice [12, 13]. The prognosis after surgical treatment is favorable, as shown by Ootaki Y et al. [4].

## Conclusions

The extremely rare subtype of the most prevalent adult congenital malformation is UCS-ASD. Although identification may pose challenges, the utilization of multimodality imaging can effectively facilitate its detection and treatment planning, as exemplified in our patient. Percutaneous interventions such as covered stenting or the implantation of an Amplatzer occluding device hold considerable potential; nevertheless, surgical repair remains the preferred primary treatment option.

## References

[1] Cinteză E-E, Filip C, Duică G, Nicolae G, Nicolescu AM, Balgradean M (2019). Unroofed coronary sinus: update on diagnosis and treatment. Rom J Morphol Embryol.

[2] Bansal RC, Martens TP, Hu H, Rabkin DG (2021). Unroofed coronary sinus discovered incidentally during cardiac surgery: systematic approach to diagnosis by transesophageal echocardiography. CASE (Phila).

[3] Awasthy N, Radhakrishnan S (2013). Stepwise evaluation of left to right shunts by echocardiography. Indian Heart J.

[4] Ootaki Y, Yamaguchi M, Yoshimura N, Oka S, Yoshida M, Hasegawa T (2003). Unroofed coronary sinus syndrome: diagnosis, classification, and surgical treatment. JTCVS.

[5] Raghib G, Ruttenberg HD, Anderson RC (1965). Termination of left superior vena cava in left atrium, atrial septal defect, and absence of coronary sinus; a developmental complex. Circulation.

[6] Quaegebeur J, Kirklin JW, Pacifico AD (1979). Surgical experience with unroofed coronary sinus. Ann Thorac Surg.

[7] Pérez Matos AJ, Planken RN, Bouma BJ (2014). Unroofed coronary sinus newly diagnosed in adult patients after corrected congenital heart disease. Neth Heart J.

[8] Ngee T, Lim MC, De Larrazabal C, Sundaram RD (2011). Unroofed coronary sinus defect. J Comput Assist Tomogr.

[9] Khadkikar G, V SM, Patel A, Shah SC, Patel TM (2021). A rare case of an unroofed coronary sinus with a persistent left superior vena cava diagnosed by two-dimensional transthoracic echocardiography. Cureus.

[10] Shi H, Yan J, Wang Q, Hua Z, Li S, Zhang J (2021). Surgical management of unroofed coronary sinus syndrome: a 20-year-single-center experience. J Card Surg.

[11] Attenhofer Jost CH, Connolly HM, Danielson GK, Dearani JA, Warnes CA, Jamil Tajik A (2007). Clinical features and surgical outcome in 25 patients with fenestrations of the coronary sinus. Cardiol Young.

[12] Torres A, Gersony WM, Hellenbrand W (2007). Closure of unroofed coronary sinus with a covered stent in a symptomatic infant. Catheter Cardiovasc Interv.

[13] Zhou Z, Gu Y, Zheng H (2022). Transcatheter closure of unroofed coronary sinus syndrome: a short-term result. Eur Heart J.

